# Mu-Opioid Receptor Agonist Induces Kir3 Currents in Mouse Peripheral Sensory Neurons – Effects of Nerve Injury

**DOI:** 10.3389/fphar.2018.01478

**Published:** 2018-12-19

**Authors:** Philip Stötzner, Viola Spahn, Melih Ö. Celik, Dominika Labuz, Halina Machelska

**Affiliations:** Department of Experimental Anesthesiology, Charité – Universitätsmedizin Berlin, Corporate Member of Freie Universität Berlin, Humboldt-Universität zu Berlin, and Berlin Institute of Health, Berlin, Germany

**Keywords:** neuropathy, DRG neurons, DAMGO, peripheral opioid receptors, potassium channels, GIRK channels, patch clamp

## Abstract

Neuropathic pain often arises from damage to peripheral nerves and is difficult to treat. Activation of opioid receptors in peripheral sensory neurons is devoid of respiratory depression, sedation, nausea, and addiction mediated in the brain, and ameliorates neuropathic pain in animal models. Mechanisms of peripheral opioid analgesia have therefore gained interest, but the role of G protein-coupled inwardly rectifying potassium (Kir3) channels, important regulators of neuronal excitability, remains unclear. Whereas functional Kir3 channels have been detected in dorsal root ganglion (DRG) neurons in rats, some studies question their contribution to opioid analgesia in inflammatory pain models in mice. However, neuropathic pain can be diminished by activation of peripheral opioid receptors in mouse models. Therefore, here we investigated effects of the selective μ-opioid receptor (MOR) agonist [D-Ala^2^, *N*-Me-Phe^4^, Gly^5^-ol]-enkephalin (DAMGO) on potassium conductance in DRG neurons upon a chronic constriction injury (CCI) of the sciatic nerve in mice. For verification, we also tested human embryonic kidney (HEK) 293 cells transfected with MOR and Kir3.2. Using patch clamp, we recorded currents at -80 mV and applied voltage ramps in high extracellular potassium concentrations, which are a highly sensitive measures of Kir3 channel activity. We found a significantly higher rate of HEK cells responding with potassium channel blocker barium-sensitive inward current (233 ± 51 pA) to DAMGO application in transfected than in untransfected group, which confirms successful recordings of inward currents through Kir3.2 channels. Interestingly, DAMGO induced similar inward currents (178 ± 36–207 ± 56 pA) in 15–20% of recorded DRG neurons from naïve mice and in 4–27% of DRG neurons from mice exposed to CCI, measured in voltage clamp or voltage ramp modes. DAMGO-induced currents in naïve and CCI groups were reversed by barium and a more selective Kir3 channel blocker tertiapin-Q. These data indicate the coupling of Kir3 channels with MOR in mouse peripheral sensory neuron cell bodies, which was unchanged after CCI. A comparative analysis of opioid-induced potassium conductance at the axonal injury site and peripheral terminals of DRG neurons could clarify the role of Kir3 channel–MOR interactions in peripheral nerve injury and opioid analgesia.

## Introduction

In European countries, 12–30% of adults suffer from chronic pain and many of them experience severe limitations in managing daily life activities (Breivik et al., 2006). Besides the impairment of patients’ quality of life, chronic pain is a major economic challenge for social and health care systems. Neuropathic pain is defined as pain caused by a lesion or disease of the somatosensory nervous system (IASP, 2017). Patients experience reduced thresholds to painful mechanical or thermal stimuli, or pain sensations elicited by normally innocuous stimuli such as touch, warm or cool temperatures (Baron et al., 2010). Common causes of this chronic condition are diabetes, herniated vertebral discs, cancer, chemotherapy, human immunodeficiency virus or varicella zoster virus, and injuries to nerves due to accidents or medical procedures (Jensen and Finnerup, 2014).

The treatment of neuropathic pain is challenging and the side effects restrict the use of many medications. Among these are opioids such as morphine and fentanyl, which are the most powerful analgesics available. However, they also induce constipation which predominantly results from activation of intestinal opioid receptors (Imam et al., 2018), as well as respiratory depression, sedation, dizziness, and nausea mediated in the central nervous system (CNS) (Li and van den Pol, 2008; Imam et al., 2018). Furthermore, their prolonged use leads to the development of addiction, which has resulted in a worldwide opioid epidemic (Volkow et al., 2018). Importantly, the activation of opioid receptors in peripheral sensory neurons can provide analgesia without CNS side effects (Stein, 1995; Kalso et al., 2002). In fact, peripheral opioid receptors mediate a large proportion of the analgesic effects produced by systemically administered opioids (Stein and Machelska, 2011; Gaveriaux-Ruff, 2013; Jagla et al., 2014; Spahn et al., 2017). Numerous preclinical studies have also shown peripheral opioid analgesia in models of neuropathic pain in rats (Truong et al., 2003; Kabli and Cahill, 2007; Obara et al., 2007, 2009; Spahn et al., 2018) and mice (Kolesnikov et al., 2007; Cayla et al., 2012; Hervera et al., 2012; Labuz and Machelska, 2013; Labuz et al., 2016). Furthermore, a clinical trial reported attenuation of neuropathic pain after peripherally applied morphine in patients (Azad et al., 2000). Activation of peripheral opioid receptors leads to the inhibition of voltage-gated calcium and sodium channels, acid-sensing ion channels, transient receptor potential cation channel subfamily V member 1 (TRPV1), and subfamily M member 3 (TRPM3) (Gold and Levine, 1996; Endres-Becker et al., 2007; Cai et al., 2014; Dembla et al., 2017). A particularly prominent mechanism underlying peripheral opioid analgesia is the activation of potassium channels in peripheral sensory neurons (Ocana et al., 2004). Hence, interactions between opioid receptors and potassium channels pose a promising basis for the development of novel therapies with an improved side effect profile.

Potassium channels play a pivotal role in the regulation of neuronal excitability and their dysregulation contributes to neuropathic pain (Prescott et al., 2014; Waxman and Zamponi, 2014). Inwardly rectifying potassium (Kir) channels have gained particular interest due to their crucial role in maintaining the resting membrane potential of neurons. Of these channels the G protein-coupled inwardly rectifying potassium (GIRK or Kir3) channels form membrane bound signaling complexes with opioid receptors (Luscher and Slesinger, 2010; Nagi and Pineyro, 2014). Four Kir3 channel subunits have been identified, Kir3.1–3.4. To form a functional channel, the subunits assemble in heterotetramers (Kir3.1, 3.3, 3.4) or both hetero- and homo-tetramers (Kir3.2). The Kir3.1–3.3 subunits are common in the CNS (Hibino et al., 2010; Luscher and Slesinger, 2010; Nagi and Pineyro, 2014). In the peripheral neuron cell bodies in dorsal root ganglia (DRG), the Kir3 mRNA and protein expression, and function have been shown in rats and humans (Khodorova et al., 2003; Gao et al., 2007; Nockemann et al., 2013; Chung et al., 2014; Gorham et al., 2014; Lyu et al., 2015). In contrast, the data on Kir3 in the mouse DRG are conflicting. Whereas some studies detected Kir3 mRNA in DRG (Manteniotis et al., 2013; Saloman et al., 2016), others did not find Kir3 mRNA or protein in DRG or cutaneous nerves in wild-type mice (Mitrovic et al., 2003; Nockemann et al., 2013). The latter study suggested that the absence of Kir3 in DRG neurons underlie the weak or absent peripheral mu-opioid receptor (MOR)-mediated analgesia in an inflammatory pain model in wild-type mice; this analgesia was established by transgenic expression of Kir3.2 in DRG neurons (Nockemann et al., 2013). However, in neuropathic pain models, opioids effectively alleviate mechanical and heat hypersensitivity via activation of peripheral MOR in wild-type mice (Kolesnikov et al., 2007; Cayla et al., 2012; Hervera et al., 2012; Labuz and Machelska, 2013; Labuz et al., 2016). These findings raise the question whether Kir3 channels functionally couple to MOR in peripheral sensory neurons in mice following neuropathy.

Therefore, our goal in this study was to investigate the effects of MOR agonist [D-Ala^2^, *N*-Me-Phe^4^, Gly^5^-ol]-enkephalin (DAMGO) on potassium conductance in DRG neurons upon peripheral nerve injury in mice. We hypothesized that the nerve lesion results in enhanced MOR-induced potassium conductance in the corresponding DRG sensory neuron cell bodies. As a model of neuropathic pain we used a chronic constriction injury (CCI) of the sciatic nerve, which resembles human peripheral neuropathy (e.g., nerve entrapment or compression) (Bennett and Xie, 1988). To this end, we examined the effects of DAMGO on potassium conductance using patch clamp in cultured DRG neurons from naïve mice and mice exposed to CCI. As a reference, equivalent experiments were performed in human embryonic kidney (HEK) 293 cells transfected with MOR and Kir3.2.

## Materials and Methods

### HEK 293 Cell Culture and Transfection

Human embryonic kidney 293 cells transfected with rat MOR and mouse Kir3.2 and untransfected HEK 293 cells (control) (German Collection of Microorganisms and Cell Cultures, Braunschweig, Germany) were maintained in DMEM (Sigma-Aldrich, Steinheim, Germany) supplemented with 1% penicillin/streptomycin and 10% fetal bovine serum (Biochrom, Berlin, Germany) in 5% CO_2_ at 37°C, and were passaged every 2–3 days. The cells were seeded onto plastic culture dishes (35 mm) a day prior to transfection. The transfection mixture consisted of 1 μg pcDNA3.1-MOR (kindly provided by Prof. Christian Zöllner), 1 μg pFLAG-Kir3.2 (kindly provided by Dr. Dinah Nockemann), 6 μl XtremeGene added to 88 μl DMEM without supplements per culture dish. Untransfected cells were cultured accordingly.

### Animals

Animal experiments were approved by the State animal care committee (Landesamt für Gesundheit und Soziales, Berlin, Germany) and followed the guidelines of the International Association for the Study of Pain (Zimmermann, 1983) and the ARRIVE guidelines (Kilkenny et al., 2010). Wild-type male C57BL/6J mice (18–35 g, 6–8 weeks old; Janvier Laboratories, France) were housed in groups of 2–4 per cage lined with ground corncob bedding, with free access to standard laboratory rodent chow and tap water, on a 12 h/12 h (8 am/8 pm) light/dark cycle. Room temperature was 22 ± 0.5°C and humidity was 60–65%.

### Chronic Constriction Injury

The CCI was induced in deeply isoflurane-anesthetized mice by exposing the sciatic nerve at the level of the right mid-thigh, and placing three loose silk ligatures (4-0) around the nerve with about 1-mm spacing. The ligatures were tied until they elicited a brief twitch in the respective hind limb. The wound was closed with silk sutures (Labuz et al., 2009; Labuz and Machelska, 2013).

### DRG Tissue Preparation and Neuron Culture

Dorsal root ganglia were isolated from naïve mice and mice 2 days after CCI. Briefly, mice were killed by an overdose of isoflurane, the vertebral column was removed, washed in PBS, placed in ice-cold PBS, and the lumbar (three to five) DRG innervating sciatic nerve ipsilateral to CCI or from the right side of naïve mice were dissected. The DRG were collected in ice-cold serum-free working medium (DMEM/HAM’s F12 supplemented with 1% penicillin/streptomycin). DRG from one animal were used for one culture. Further handling of the tissue was performed under a laminar air flow hood in sterile conditions. The collected DRG tissue was incubated in 1.25% collagenase (Sigma-Aldrich) for 50 min at 37°C in a thermoshaker, washed with PBS and incubated in 2.5% trypsin (Sigma-Aldrich) for 5 min at 37°C in a thermoshaker. After digestion, the tissue was triturated using plastic pipette tips and subsequently filtered through a 40-μm cell strainer. The filtrate was centrifuged, the supernatant discarded and the cell pellet resuspended in 300–1000 μl culture medium (DMEM/HAM’s F12 supplemented with 1% penicillin/streptomycin and 10% horse serum), depending on the required cell density. The cell suspensions (30–100 μl) were then seeded onto poly-L-lysine coated plastic culture dishes (35 mm) and placed in an incubator (5% CO_2_ at 37°C). An hour later (to allow the cells to settle down), the cell cultures were topped up to a total of 2 ml of culture medium and cultured until patch clamp recordings, as previously described (Nockemann et al., 2013).

### Patch Clamp Experiments

Human embryonic kidney 293 cells were recorded 40–50 h after plating (untransfected cells) or transfection with MOR and Kir3.2. DRG neurons (medium diameter of 20–35 μm) (Stucky and Lewin, 1999) were used 20–30 h after cultivation. Cell viability was evaluated before first experiment by Trypan Blue exclusion assay. Recordings were carried out in whole-cell voltage clamp mode. After washing with PBS, cells were bathed in low potassium extracellular buffer (5.6 mM KCl, 140 mM NaCl, 2.6 mM CaCl_2_, 1.2 mM MgCl_2_, 10 mM HEPES, 2.6 mM glucose; adjusted to pH 7.4 using NaOH; all from Sigma-Aldrich) and visualized using a Zeiss Axiovert 200 inverse microscope. Patch pipettes (resistance 3.5–8 MΩ) were fabricated from Borosilicate glass capillaries using a Sutter P-97 puller (Sutter Instrument, Novato, CA, United States) and filled with intracellular buffer (122 mM KCl, 5 mM NaCl, 1 mM CaCl_2_, 2 mM MgCl_2_, 10 mM HEPES, 11 mM EGTA, 4 mM MgATP, 0.25 mM NaGTP; adjusted to pH 7.4 using KOH; all from Sigma-Aldrich). Currents were amplified and recorded using an EPC-10 patch amplifier and Pulse software (HEKA, Lambrecht, Germany), and were sampled at a frequency of 100 Hz. Cells were superfused by steady flow of extracellular buffer at a flow rate of 800–1000 μl/min using a pressurized application system (Perfusion Pressure Kit VPP-6; Warner Instruments, Hamden, CT, United States) and a suction pump. Test compounds, DAMGO (10 μM), BaCl_2_ (3 mM; both from Sigma-Aldrich), and tertiapin-Q (100 nM; Alomone Labs, Jerusalem, Israel) were applied using a perfusion valve control systems (VC-6; Warner Instruments) to switch between vehicle buffer and buffers containing the test compounds. All recordings were performed at room temperature. Fast capacitive currents (i.e., pipette potential) were canceled before seal formation. After reaching “giga-seal,” the membrane patch was ruptured to achieve whole-cell configuration. In DRG neurons, the resting membrane potential was estimated in current-clamp mode shortly after gaining whole-cell access and action potentials were recorded in current-clamp mode using stepwise increasing current injections of 100 ms from 100 to 600 pA. Only cells showing proper action potential overshoot were included for further experiments. Cell capacitance, series and input resistance were monitored by applying test pulses of 10 mV for 10 ms before each recording. The currents were recorded in voltage-clamp mode at a constant holding potential of -80 mV in high potassium buffer (140 mM KCl, 2.6 mM CaCl_2_, 1.2 mM MgCl_2_, 5 mM HEPES; adjusted to pH 7.4 using KOH) for 120 s in the absence or presence of DAMGO without or with BaCl_2_ (Nockemann et al., 2013). Hyperpolarized state and high concentration of potassium in extracellular buffer were used to increase the electro-chemical gradient for potassium to drive it into the cell when Kir3 channels are opened. To reduce “stress” to the cells, high potassium buffer was carefully washed in over a period of 2 min and cells were allowed to stabilize for at least 2 min before recording. For tertiapin-Q experiments we applied voltage ramps from a holding potential of -40 mV and measured the induced current at -80 mV, based on previously published protocols (Gao et al., 2007; Gorham et al., 2014). These experiments were performed in a 45 mM high potassium extracellular buffer (45 mM KCl, 100 mM NaCl, 2 mM CaCl_2_, 1 mM MgCl_2_, 10 mM HEPES, 10 mM glucose; adjusted to pH 7.4 using KOH) and an intracellular solution consisting of 120 mM KCl, 20 mM NaCl, 3 mM MgCl_2_, 10 mM HEPES, 10 mM glucose, 1 mM EGTA, 3 mM NaATP, 0.3 mM NaGTP (adjusted to pH 7.4 using KOH) (Gorham et al., 2014). The ramps were applied every 10 s for 200 s in the absence or presence of DAMGO without or with tertiapin-Q. The analysis of patch clamp recordings was performed using Nest-o-Patch v1.2 and Prism v6 software (GraphPad Software, Inc., La Jolla, CA, United States). Effects of DAMGO were measured as departure from holding current (in voltage clamp mode experiments) or baseline currents (in voltage ramp experiments) while running vehicle buffer. Cells were considered responding to DAMGO application (DAMGO-responders) if the resulting current was larger than three times the noise range from the holding and baseline currents, respectively. Effects of BaCl_2_ were measured as departure from holding current while running DAMGO buffer. Effects of tertiapin-Q were measured as departure from the mean baseline current while running DAMGO buffer. Drift of baseline was corrected manually or using the Nest-o-Patch baseline correction tool when necessary.

### Statistical Analyses

Data are shown in raw values as bars representing cell numbers, representative currents, individual data points representing single cell currents, or means ± SEM. The number of cells per group was 13–41; the exact numbers are given in Figures. Statistical analyses were performed using GraphPad Prism software (Version 5.02 for Windows; GraphPad Software, Inc.). The data were normally distributed as evaluated by Kolmogorov–Smirnov test. The comparison of DAMGO-induced currents between HEK 293 cells and DRG neurons was done by one-way analysis of variance (ANOVA). The comparison of DAMGO-induced currents between DRG neurons from naïve and CCI mice was analyzed by unpaired *t*-test. The reversibility of DAMGO-induced currents by BaCl_2_ or tertiapin-Q was assessed by paired *t*-test. To compare ratios of DAMGO-responders to DAMGO-non-responders between naïve and CCI mice as well as between untransfected and transfected HEK 293 cells, the Fisher’s exact test was used. The differences were defined as statistically significant if *P* < 0.05. The statistical tests and the degree of significance are specified in the Section “Results” or figure captions.

## Results

### DAMGO Induces Potassium Currents in HEK 293 Cells Transfected With MOR and Kir3.2

**FIGURE 1:**
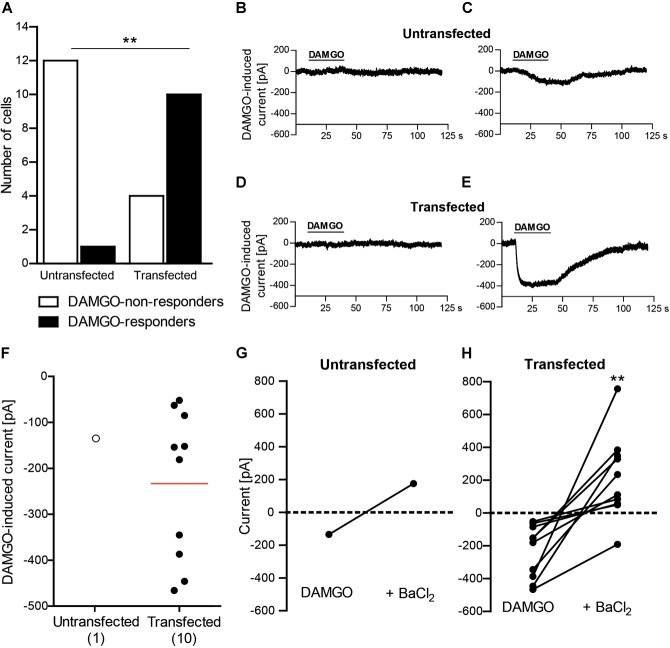
DAMGO (10 μM) induces potassium currents in HEK 293 cells transfected with rat MOR and mouse Kir3.2. **(A)** Number of DAMGO-responders and non-responders in untransfected and transfected cells. ^∗∗^*P* = 0.0013 (Fisher’s exact test) indicates higher proportion of DAMGO-responders to DAMGO-non-responders in transfected vs. untransfected cells. The cells were sampled from three untransfected and four transfected cell cultures. **(B–E)** Exemplary traces of DAMGO-non-responder **(B)** and DAMGO-responder **(C)** in untransfected cells, and of DAMGO-non-responder **(D)** and DAMGO-responder **(E)** in transfected cells. **(F)** Single cell currents in DAMGO-responders. The data points represent single cell values, and the red horizontal line indicates the mean. Numbers in brackets indicate the number of cells. **(G,H)** BaCl_2_ (3 mM)-mediated reversibility of DAMGO-induced currents in untransfected cells (*n* = 1) **(G)** and transfected cells (*n* = 10; ^∗∗^*P* = 0.002, paired *t*-test) **(H)**. Only DAMGO-responders are shown. Data points represent DAMGO-induced currents of the same cell before and after application of BaCl_2_. Dotted lines represent zero current. In all experiments, the currents were recorded in voltage clamp mode at –80 mV in high potassium extracellular buffer (140 mM). Cells were defined as responding to DAMGO if the resulting current was larger than three times the noise range.

To establish the protocol for patch clamp recordings of inward potassium currents, we first used HEK 293 cells transfected with MOR and Kir3.2 and untransfected (control) HEK 293 cells. Effects of DAMGO (10 μM) were recorded in the whole-cell voltage clamp mode at constant holding potential of -80 mV and high potassium extracellular buffer (140 mM). Analysis of all recorded HEK 293 cells revealed a significantly higher rate of cells responding with inward current to DAMGO application (DAMGO-responders) in transfected than in untransfected group (Figure 1A). In the untransfected group, vast majority of cells did not respond to DAMGO (92%; 12 of total 13 recorded cells) (Figures 1A,B), and only one cell showed very small, questionable response to DAMGO (Figures 1A,C) (see also section “Discussion”). In contrast, most of the cells in the transfected group showed prompt and reversible (by washout) inward currents upon DAMGO application (71%; 10 of total 14 recoded cells), whereas four cells (29%) were classified as DAMGO-non-responders (Figures 1A,D,E). The currents were similar in all DAMGO-responders, although statistical analysis could not be performed as there was only one responder among untransfected cells (Figure 1F). Application of the potassium channel blocker barium (3 mM BaCl_2_) reversed DAMGO-mediated currents in one untransfected cell responding to DAMGO (Figure 1G) and in all DAMGO-responding transfected cells (Figure 1H), indicating that inward currents were mediated by potassium channels. These results clearly show functional coupling of MOR and Kir3.2 in transfected HEK 293 cells.

### DAMGO Induces Potassium Currents in Mouse DRG Neurons

**FIGURE 2:**
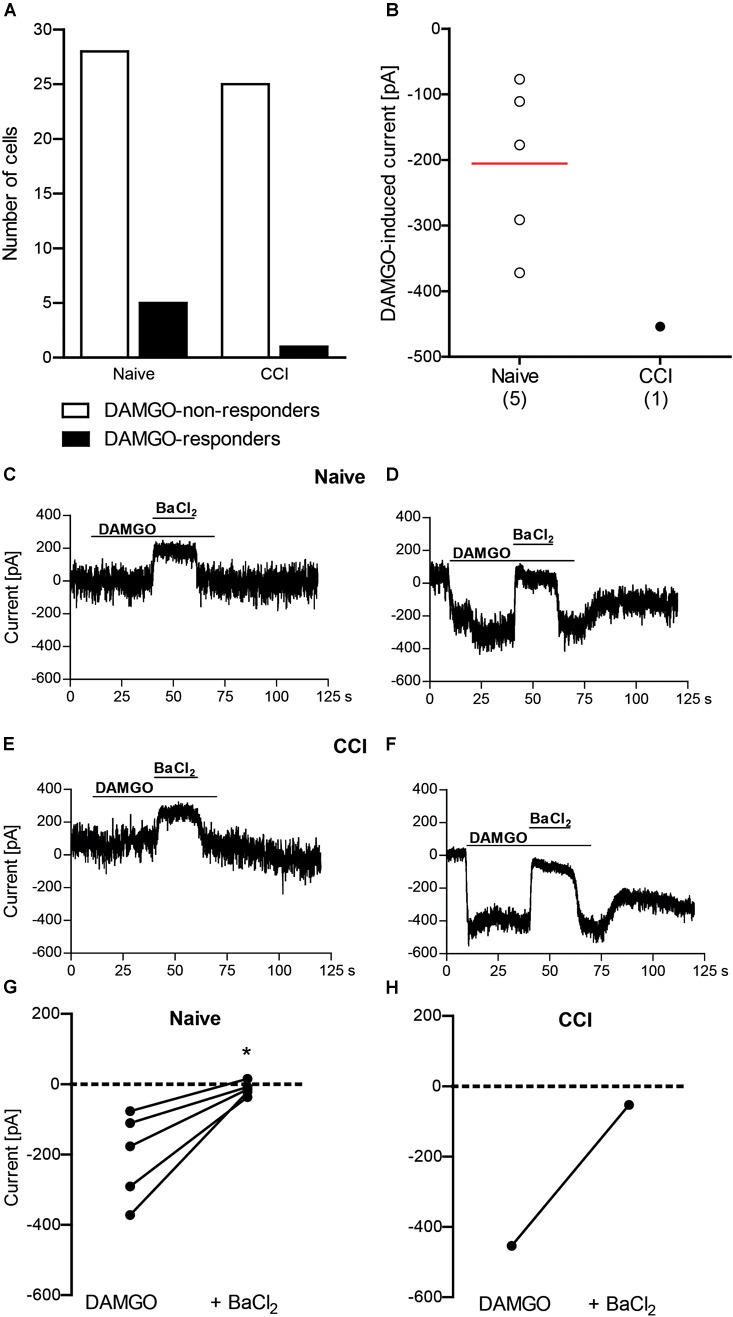
DAMGO (10 μM) induces potassium currents in mouse DRG neurons, assessed in the voltage clamp mode. **(A)** Number of neurons responding and non-responding to DAMGO from naïve and CCI mice. The proportion of DAMGO-responding to DAMGO-non-responding neurons from naïve vs. CCI mice did not differ significantly (*P* = 0.2148; Fisher’s exact *t*-test). The neurons were sampled from cultures obtained from DRG of nine naïve mice and eight CCI mice. **(B)** Single neuron currents in DAMGO-responders. The data points represent single neuron values, and the red horizontal line indicates the mean. Numbers in brackets indicate (the number of neurons. **(C–F)** Exemplary traces of DRG neurons non-responding **(C)** and responding **(D)** to DAMGO from naïve mice, and DRG neurons non-responding **(E)** and responding **(F)** to DAMGO from mice on day 2 following CCI. The DAMGO effects are shown before and during BaCl_2_ (3 mM) application. **(G,H)** BaCl_2_ (3 mM)-mediated reversibility of DAMGO-induced currents in individual neurons from naïve mice (*n* = 5 neurons; ^∗^*P* = 0.017, paired *t*-test) **(G)** and CCI mice (*n* = 1 neuron) **(H)**. Only DAMGO-responding neurons are shown. Data points represent DAMGO-induced currents of the same neuron before and after application of BaCl_2_. Dotted lines represent zero current. In all experiments, the currents were recorded in voltage clamp mode at –80 mV in high potassium extracellular buffer (140 mM). Neurons were defined as responding to DAMGO if the resulting current was larger than three times the noise range.)

**FIGURE 3:**
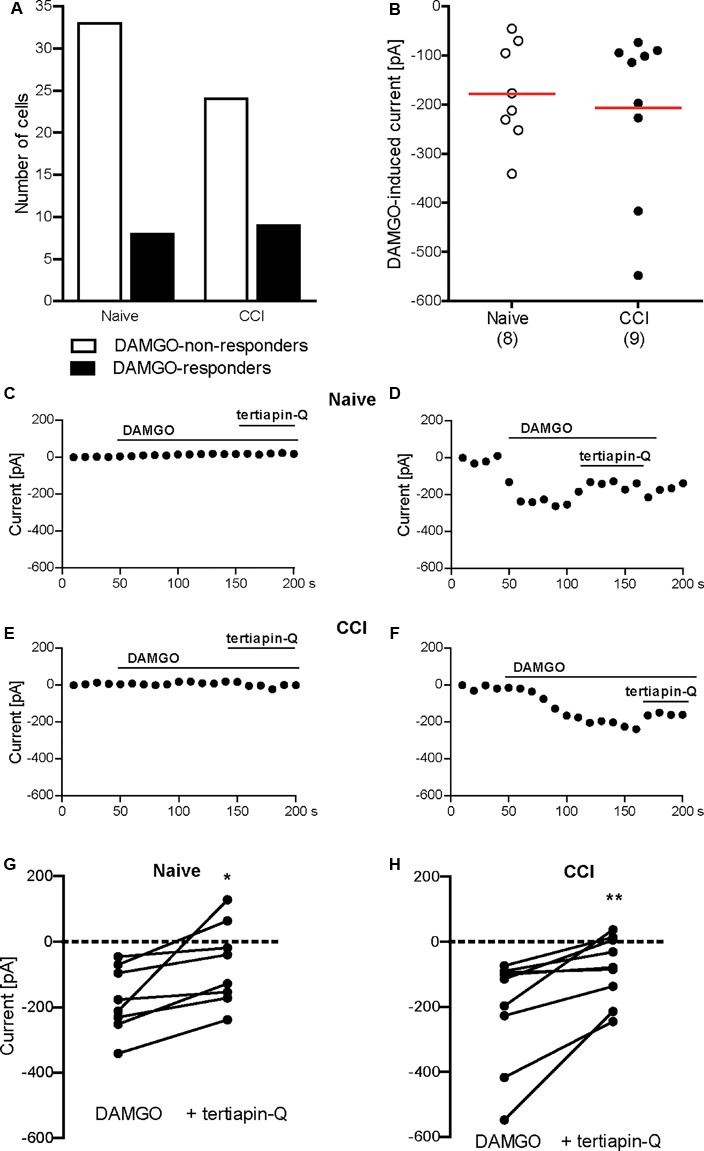
DAMGO (10 μM)-induced potassium currents in mouse DRG neurons obtained in the voltage ramp mode. **(A)** Number of neurons responding and non-responding to DAMGO from naïve and CCI mice. The proportion of DAMGO-responding to DAMGO-non-responding neurons from naïve vs. CCI mice did not differ significantly (*P* = 0.596; Fisher’s exact *t*-test). The neurons were sampled from cultures obtained from DRG of seven naïve and eight CCI mice. **(B)** Single neuron currents in DAMGO-responders. The data points represent single neuron values, and the red horizontal lines indicate the means. Numbers in brackets indicate the number of neurons. **(C–F)** Exemplary currents of DRG neurons non-responding **(C)** and responding **(D)** to DAMGO from naïve mice, and DRG neurons non-responding **(E)** and responding **(F)** to DAMGO from mice on day 2 following CCI. The DAMGO effects are shown before and during tertiapin-Q (100 nM) application. **(G,H)** Tertiapin-Q (100 nM)-mediated attenuation of DAMGO-induced currents in individual neurons from naïve mice (*n* = 8 neurons; ^∗^*P* = 0.0204, paired *t*-test) **(G)** and CCI mice (*n* = 9 neurons; ^∗∗^*P* = 0.0073, paired *t*-test) **(H)**. Only DAMGO-responding neurons are shown. Data points represent DAMGO-induced currents of the same neuron before and after application of tertiapin-Q. Dotted lines represent zero current. In all experiments, the currents were obtained by voltage ramps from a holding potential of –40 mV and measured at –80 mV in high potassium extracellular buffer (45 mM). Neurons were defined as responding to DAMGO if the resulting current was larger than three times the noise range.

Conferring the recording conditions established for HEK 293 cells, in the next set of experiments we investigated the effects of nerve injury on DAMGO (10 μM)-induced potassium conductance in mouse DRG neurons. In addition, we used voltage ramp experiments in 45 mM potassium extracellular buffer to reduce the ionic stress in tertiapin-Q experiments, since the viability of the neurons dramatically decreased during tertiapin-Q application in voltage clamp mode in 140 mM potassium buffer. We analyzed neurons from DRG of naïve mice and from DRG ipsilateral to the CCI (2 days after CCI). In previous studies, we have shown that mechanical and heat hypersensitivities are fully established and can be attenuated by peripherally applied DAMGO at this time point following CCI in mice *in vivo* (Cayla et al., 2012; Labuz and Machelska, 2013; Labuz et al., 2016). Here we found that the rate of DAMGO-responding neurons between naïve and CCI mice was not significantly different (Figures 2A, 3A). Thus, in the voltage clamp mode experiments (Figure 2A), we recorded 15% DAMGO-responders (5 of total 33 recorded neurons) from naïve mice and 4% DAMGO-responders (1 of total 26 recorded neurons) from mice exposed to CCI. In voltage ramp experiments (Figure 3A), we recorded 20% DAMGO-responders (8 of total 41 recorded neurons) from naïve mice and 27% DAMGO-responders (9 of total 33 recorded neurons) from CCI mice. DAMGO-induced inward currents were comparable between neurons from naïve and CCI mice; due to low number of DAMGO-responders in the voltage clamp mode (Figure 2B), the statistical analysis could only be performed for the voltage ramp experiments (Figure 3B) (*P* = 0.6781, unpaired *t*-test). Nevertheless, in both experimental conditions the DAMGO-responding neurons showed prompt inward currents, which could be reversed by barium (3 mM BaCl_2_) (Figures 2C–H) and attenuated by a more selective Kir3 channel blocker tertiapin-Q (100 nM) (Figures 3C–H). Additionally, the currents of DAMGO-responding neurons from naïve mice (Figures 2B, 3B) and CCI mice (Figure 3B) were comparable to currents of DAMGO-responders in MOR- and Kir3.2-transfected HEK 293 cells (Figure 1F) (*P* = 0.8866, one-way ANOVA).

## Discussion

In this study, we found that the MOR-selective agonist DAMGO induces potassium currents in DRG neurons of both naïve mice and mice with CCI of the sciatic nerve, which were diminished by barium and tertiapin-Q indicating the involvement of Kir3 channels. The rate of DAMGO-responding neurons and the DAMGO-induced inward currents did not change following CCI.

In initial experiments, we determined DAMGO-induced inward potassium currents using a whole-cell voltage clamp approach in hyperpolarized untransfected and transfected with MOR and Kir3.2 HEK 293 cells. Among the untransfected cells, one cell was classified as DAMGO-responder according to the criterion that the DAMGO-mediated current is three times larger than the noise range. However, based on the response kinetics (slow and not clearly corresponding to DAMGO application, very small current amplitude; Figure 1C), this cell did not appear to reliably respond to DAMGO. In contrast, the response of transfected cells was fast and had distinct and tightly correlated to DAMGO application onset (Figure 1E), similar to the literature (Kohno et al., 2005; Kobayashi et al., 2006; Nockemann et al., 2013; Gorham et al., 2014). Furthermore, substantially higher ratio of DAMGO-responders in MOR- and Kir3.2-transfected compared to untransfected HEK 293 cells, and the reversibility of DAMGO-induced currents by potassium channel blocker barium clearly demonstrate successful identification of DAMGO-induced inward currents and suggest they were mediated by Kir3.2 channels.

Our finding that DAMGO induced similar currents in DRG neurons of naïve wild-type mice is somewhat intriguing. Whereas functional Kir3 channels have been consistently identified in rat peripheral sensory neurons (Gao et al., 2007; Nockemann et al., 2013; Chung et al., 2014), only a few studies investigated Kir3 channels in these neurons in mice, and the data are inconsistent. Nockemann et al. (2013) showed very low amounts of Kir3.1 and Kir3.2 mRNA transcripts and no immunoreactivity of the corresponding proteins in mouse DRG. Using patch clamp recordings, the authors reported “negligible” inward currents upon DAMGO application and concluded on the absence of Kir3 from mouse DRG neurons. However, the size of DAMGO-induced currents they measured in naïve wild-type mouse DRG neurons are substantial (1.8 ± 0.4 nA) (Nockemann et al., 2013) and in fact, much higher compared to currents recorded under similar conditions and defined as opioid-mediated responses in rat DRG and spinal cord neurons, or in Xenopus oocytes transfected with Kir3 (40–800 pA) in other studies (Kohno et al., 2005; Kobayashi et al., 2006; Gao et al., 2007). The results in these latter publications are indeed similar to our recordings in MOR- and Kir3.2-transfected HEK 293 cells (233 ± 51 pA; Figure 1F), and DRG neurons from naïve mice (206 ± 55 pA, Figure 2B; 178 ± 36 pA, Figure 3B) and CCI mice (207 ± 56 pA, Figure 3B). Kanjhan et al. (2005) reported the absence of hyperpolarization-activated potassium currents characteristic for Kir3 in DRG neurons of newborn mice and argued that Kir3 expression might occur later in the development of the nervous system, but did not examine older animals. Mitrovic et al. (2003) stated a lack of Kir3.2-immunostaining in mouse DRG, but did not present the corresponding data. In a comprehensive RNA expression analysis of mouse sensory ganglia, Manteniotis et al. (2013) reported moderate levels of KCNJ3 mRNA coding for Kir3.1 in DRG and trigeminal ganglia. This, however, is insufficient to form a functional channel, since functional Kir3 channels are formed by Kir3.1/Kir3.2 heterotetramers and Kir3.2 homotetramers (Luscher and Slesinger, 2010). Interestingly, a recent study found mRNAs encoding Kir3.1 and Kir3.2 in mouse DRG, which would allow formation of functional Kir3 channels (Saloman et al., 2016). Taken together, the current literature suggests low to moderate Kir3 mRNA expression (Nockemann et al., 2013; Saloman et al., 2016), which may result in low protein level difficult to detect by immunostaining (Mitrovic et al., 2003; Nockemann et al., 2013) and functional analysis (Nockemann et al., 2013) in mouse DRG neurons. Yet, we found a moderate number of neurons (15–20%) reliably responding to DAMGO with inward currents in DRG of naïve wild-type mice. DAMGO-induced currents in our experiments were diminished by both a general potassium channel blocker barium, and by tertiapin-Q, currently considered the most selective Kir3 channel blocker (Jin and Lu, 1998; Kitamura et al., 2000). Furthermore, considering the patch clamp conditions in our experiments such as high potassium concentration in the extracellular buffer and hyperpolarizing holding potential or voltage ramp mode, which are highly sensitive measures of Kir3 channel activity (Kobayashi et al., 2006; Gao et al., 2007; Nockemann et al., 2013; Gorham et al., 2014), the DAMGO-mediated potassium currents in mouse DRG neurons in our experiments likely resulted from the activation of Kir3 channels. This is also supported by the finding that currents of DAMGO-responding neurons were comparable to Kir3 currents measured in our MOR- and Kir3.2-transfected HEK 293 cell experiments, and in rat neurons and Xenopus oocytes in other studies (Kohno et al., 2005; Kobayashi et al., 2006; Gao et al., 2007). Although we have not used MOR antagonist, DAMGO is the MOR-selective agonist (Labuz and Machelska, 2013), and its effects in the dose of 10 μM (we used here) were reversible by opioid receptor antagonist naloxone in patch clamp experiments (Nockemann et al., 2013), suggesting that DAMGO-induced potassium currents in our experiments are MOR-mediated.

We have also analyzed DRG neurons from mice following sciatic nerve CCI, and found one DAMGO-responding neuron (i.e., 4% of all recorded neurons) in the voltage clamp mode, and nine DAMGO-responding neurons (i.e., 27% of all recorded neurons) in the voltage ramp experiments. This was not significantly different compared to DAMGO-responding neurons from naïve mice (15% and 20%, respectively). Also the DAMGO-induced inward currents were comparable between neurons from naïve and CCI mice (see paragraph above and Figure 3B). As such functional analysis following CCI has not been performed previously, several studies examined expression of Kir3 channels or MOR. Following CCI of the sciatic nerve in transgenic Kir3.2 mice, the Kir3.2 mRNA in the DRG was not altered (Nockemann et al., 2013). The MOR mRNA or protein levels were either decreased (Obara et al., 2009), not altered (Briscini et al., 2002; Kolesnikov et al., 2007), or elevated (Truong et al., 2003) following CCI. Regardless of these effects in the DRG cell bodies, the activation of MOR on DRG neuron peripheral terminals consistently attenuated CCI-induced hypersensitivity *in vivo* and nociceptor excitability *ex vivo* in mice (Kolesnikov et al., 2007; Cayla et al., 2012; Hervera et al., 2012; Schmidt et al., 2012; Labuz and Machelska, 2013; Labuz et al., 2016). This is in line with the accumulation of Kir3.1, Kir3.2, and MOR proteins at the site of nerve ligation (Schmidt et al., 2013; Lyu et al., 2015) and the alleviation of CCI-induced hypersensitivity by DAMGO applied at the CCI site (Cayla et al., 2012; Labuz and Machelska, 2013; Labuz et al., 2016). Therefore, the corresponding electrophysiological recordings from the injury site, for example using *in vitro* skin-nerve preparations, appear appealing, but are technically very challenging.

## Conclusion and Relevance

Using electrophysiology, we addressed here for the first time the effect of MOR agonist DAMGO on potassium currents in mouse peripheral neurons following CCI of the sciatic nerve. Our data indicate a coupling of MOR and Kir3 in DRG neurons in naïve mice and following CCI. The number of responding neurons and the size of DAMGO-induced potassium currents were comparable between both groups. Hence, the MOR–Kir3 interactions in peripheral sensory neurons in attenuation of neuropathic pain presents a worthwhile target for further investigations. Particularly, site-specific analysis of opioid-mediated Kir3 conductance along the peripheral pain pathway, including injury site at the axon and peripheral terminals, could elucidate the role of Kir3 and MOR in peripheral neuropathies and their alleviation.

## Author Contributions

PS designed and performed the experiments, analyzed and interpreted the results, and wrote the manuscript. VS designed and performed the experiments, analyzed and interpreted the results, and wrote the manuscript. MÖC and DL performed animal surgeries. HM conceptualized the project, designed experiments, interpreted the results, and wrote the manuscript. All authors accepted the final version of the manuscript.

## Conflict of Interest Statement

The authors declare that the research was conducted in the absence of any commercial or financial relationships that could be construed as a potential conflict of interest.
